# Detection of Co-expressed Pathway Modules Associated With Mineral Concentration and Meat Quality in Nelore Cattle

**DOI:** 10.3389/fgene.2019.00210

**Published:** 2019-03-13

**Authors:** Wellison J. S. Diniz, Gianluca Mazzoni, Luiz L. Coutinho, Priyanka Banerjee, Ludwig Geistlinger, Aline S. M. Cesar, Francesca Bertolini, Juliana Afonso, Priscila S. N. de Oliveira, Polyana C. Tizioto, Haja N. Kadarmideen, Luciana C. A. Regitano

**Affiliations:** ^1^Department of Genetics and Evolution, Federal University of São Carlos, São Carlos, Brazil; ^2^Department of Applied Mathematics and Computer Science, Technical University of Denmark, Kongens Lyngby, Denmark; ^3^Embrapa Pecuária Sudeste, Empresa Brasileira de Pesquisa Agropecuária, São Paulo, Brazil; ^4^Department of Health Technology, Technical University of Denmark, Kongens Lyngby, Denmark; ^5^Department of Animal Science, Luiz de Queiroz College of Agriculture, University of São Paulo, Piracicaba, Brazil; ^6^Graduate School of Public Health and Health Policy, The City University of New York, New York, NY, United States; ^7^Department of Aquaculture, Technical University of Denmark, Kongens Lyngby, Denmark

**Keywords:** AMPK pathway, co-expression analysis, intramuscular fat, RNA sequencing, tenderness

## Abstract

Meat quality is a complex trait that is influenced by genetic and environmental factors, which includes mineral concentration. However, the association between mineral concentration and meat quality, and the specific molecular pathways underlying this association, are not well explored. We therefore analyzed gene expression as measured with RNA-seq in *Longissimus thoracis* muscle of 194 Nelore steers for association with three meat quality traits (intramuscular fat, meat pH, and tenderness) and the concentration of 13 minerals (Ca, Cr, Co, Cu, Fe, K, Mg, Mn, Na, P, S, Se, and Zn). We identified seven sets of co-expressed genes (modules) associated with at least two traits, which indicates that common pathways influence these traits. From pathway analysis of module hub genes, we further found an over-representation for energy and protein metabolism (AMPK and mTOR signaling pathways) in addition to muscle growth, and protein turnover pathways. Among the identified hub genes *FASN, ELOV5*, and *PDE3B* are involved with lipid metabolism and were affected by previously identified eQTLs associated to fat deposition. The reported hub genes and over-represented pathways provide evidence of interplay among gene expression, mineral concentration, and meat quality traits. Future studies investigating the effect of different levels of mineral supplementation in the gene expression and meat quality traits could help us to elucidate the regulatory mechanism by which the genes/pathways are affected.

## Introduction

Meat is an important source of nutrients in the human diet. Meat quality traits such as intramuscular fat content (IMF), mineral concentration, and fatty acid profile influence consumer purchase decision (Ahlberg et al., 2014; Mateescu, 2014) and human health (Pighin et al., 2016). Mineral deficiency, mainly iron and zinc (Ritchie and Roser, 2018), and protein deficiency (Clugston and Smith, 2002), have been reported as worldwide health hazards. In addition, IMF, meat pH, and muscle mineral concentration also affect meat tenderness, flavor, and juiciness, which are major sensory traits related to eat satisfaction (Engle et al., 2000; Ahlberg et al., 2014; Pannier et al., 2014).

Brazil is one of the largest exporters of meat and meat products, and the Brazilian cattle herd is mainly composed of Nelore and its crosses (ABIEC, 2018). Despite being well adapted to tropical climate, Nelore cattle has typically less tender and marble meat when compared with European breeds due to several genetic and environmental factors (Cesar et al., 2015; Tizioto et al., 2015). Genome-wide association (GWAS) of SNPs (Tizioto et al., 2013, 2015; Cesar et al., 2014) and copy-number variations (CNVs) (Silva et al., 2016) in conjunction with transcriptomic studies (Diniz et al., 2016; Silva-Vignato et al., 2017; Geistlinger et al., 2018; Gonçalves et al., 2018), have illustrated the genetic factors affecting complex traits in Nelore. However, growing evidence suggested interplay among gene expression, mineral concentration, and meat quality traits, which are still unclear.

Multi-omic data integration has been useful to reveal potential causal and regulatory mechanisms underlying complex animal production, reproduction and welfare traits [reviewed in Suravajhala et al. (2016)]. Integrating genomic, transcriptomic, and phenotype data has contributed to an improved understanding of complex traits by identifying regulatory candidate genes and biological functions (Ponsuksili et al., 2013; Cesar et al., 2018; Geistlinger et al., 2018; Gonçalves et al., 2018). Based on that, Mateescu et al. (2017) carried out a GWAS combined with gene network analysis for association with the carcass, meat quality traits and mineral concentration. Among the identified pathways, the authors pointed out calcium-related processes, apoptosis, and TGF-beta signaling involved with these traits.

Genome-wide association and differential gene expression analyses have been fruitful in investigating the role of genes in complex phenotypes. However, biological systems are a result of complex interactions among genes and multiple regulatory mechanisms, which are not explored in the above-mentioned studies. To address the relationship between transcriptome and traits, co-expression networks have been successfully employed. This approach allows to identify and cluster highly connected genes and associate them to the phenotypes, shedding light on the common pathways underlying these traits as well as the main regulators (Langfelder and Horvath, 2008). To date, there is no information about this approach integrating meat quality traits and mineral concentration in beef cattle. In addition, we still have a lack of knowledge about the interplay among gene expression, mineral concentration, and meat quality traits. Thus, to explore regulatory pathways, putative gene regulators, and to study their relationship with muscle and mineral metabolism in Nelore skeletal muscle, we integrated gene expression, eQTL variation, mineral concentration (macro and micro minerals), and meat quality traits (intramuscular fat, shear force, and meat pH) based on a network approach.

## Materials and Methods

### Ethics Statement

The Institutional Animal Care and Use Committee (IACUC) from the Empresa Brasileira de Pesquisa Agropecuária (EMBRAPA – Pecuária Sudeste) approved all experimental procedures involving the animals used in this study.

### Animals and Phenotyping

A total of two hundred Nelore steers (produced at Embrapa Pecuária Sudeste, São Carlos – Brazil) were used in this study. The experimental design, production system, and animal management were previously described (Tizioto et al., 2015; Diniz et al., 2016). Briefly, animals were raised in the grazing system until 21 months of age when they were taken to three feedlots under similar nutritional and sanitary management. The Nelore steers with an average age of 25 months were harvested at commercial facilities after about 90 days of feeding and the *Longissimus thoracis* (LT) muscle samples were collected.

The steaks (2.5 cm) harvested as a cross-section of the LT muscle (11th and 13th ribs) collected at slaughter were used to measure the beef quality traits as described (Tizioto et al., 2013; Cesar et al., 2014). The traits evaluated were tenderness (Warner-Bratzler shear force – WBSF7, kg) measured 7 days after slaughter, meat pH measured 24 h after slaughter along with intramuscular fat (IMF%) (Tizioto et al., 2013).

Tissue samples were used for total RNA extraction (Diniz et al., 2016) and mineral measurement (Tizioto et al., 2014). The concentration of macro minerals [calcium (Ca), magnesium (Mg), phosphorus (P), potassium (K), sodium (Na), sulfur (S)] and micro minerals [chromium (Cr), cobalt (Co), copper (Cu), manganese (Mn), selenium (Se), iron (Fe), and zinc (Zn)] were measured using inductively coupled plasma-optical emission spectrometry (ICP OES; Vista Pro-CCD ICP OES1, radial view, Varian, Mulgrave, Australia) as described by Tizioto et al. (2014).

### Genome Expression Profile, Sequencing, and Data Processing

The LT muscle samples were collected immediately after slaughter, snap frozen in liquid nitrogen and kept at -80°C until RNA extraction. To extract RNA, approximately 100 mg of frozen tissue was used, and total RNA was purified using Trizol^®^ standard protocol (Life Technologies, Carlsbad, CA, United States). The mRNA concentration and quality were evaluated in the Bioanalyzer 2100^®^ (Agilent, Santa Clara, CA, United States).

The Illumina TruSeq^®^ RNA Sample Preparation Kit v2 Guide (San Diego, CA, United States) protocol was used to generate cDNA libraries for each sample using 2 μg of total RNA as input. Library preparation and sequencing were conducted by ESALQ Genomics Center (Piracicaba, São Paulo, Brazil). cDNA libraries were purified and validated using Agilent 2100 Bioanalyzer (Santa Clara, CA, United States). Paired-end (PE) sequencing was performed on Illumina Hiseq 2500^®^ (San Diego, CA, United States) platform following the standard protocols. The samples were multiplexed and run on multiple lanes to obtain 2 × 100 bp reads.

The PE reads were filtered using the Seqyclean package version 1.4.13 (^1^Zhbannikov et al., 2017), which removed all reads with a mean quality under 24, length under 65 bp, as well as the adapter sequences. Quality control (QC) of raw RNA-Seq reads was carried out with FastQC version 0.11.2 (^2^Andrews, 2010) and MultiQC version1.4 (^3^Ewels et al., 2016).

Read mapping and gene counting were carried out by STAR aligner version 2.5.4b (Dobin et al., 2013) using a reference genome (*Bos taurus*, ARS-UCD1.2) and gene annotation file (release 106) obtained from NCBI (NCBI, 2018). One sample with mapping rate lower than 70% was removed out for further analyses.

The data editing was done using the Bioconductor package edgeR version 3.20.9 (Robinson et al., 2010). Taking into account that low expressed genes are less reliable and indistinguishable from sampling noise (Tarazona et al., 2015), the read counts per gene were normalized to counts per million (*cpm* function). The genes with less than one *cpm* in more than 90% of the samples were filtered out. Gene counts were normalized applying the variance stabilizing transformation (*VST*) from DESeq2 version 1.18.1 (Anders and Huber, 2010).

Potential biases due to technical variation in gene expression among samples were evaluated by applying a Principal Component Analysis (PCA) and hierarchical clustering on normalized data using NOISeq version 2.22.1 (Tarazona et al., 2015). A linear model was fitted in order to adjust the gene expression matrix for batch effect (flow cell). To this end, the *removeBatchEffect* function from Limma (version 3.34.9) R package (Ritchie et al., 2015) was used. Three samples were identified as outliers. Thus, 12 known housekeeping genes were selected based on the literature (*ACTB, API5, EIF2B2, GAPDH, GUSB, HMBS, PGK1, PPIA, RPL13A, VAPB, YWHAZ*) to evaluate their variability on the samples. The housekeeping genes expression confirmed these samples as outliers, and therefore, they were filtered out.

### Network Gene Co-expression Analysis

A co-expression approach was applied using the WGCNA R package version 1.63 (Langfelder and Horvath, 2008). The method adopted for constructing the networks included two steps: First, a similarity co-expression network was calculated with Pearson’s correlation for all genes, followed by transformation to a signed adjacency matrix (AM) by using the soft thresholding power β, to which co-expression similarity is raised. Based on the criteria of approximating scale-free topology, we chose the power of β = 12 such that the resulting network satisfies the scale-free topology (linear regression model fitting index *R^2^* = 0.80).

Outlier animals (*n* = 2) were identified based on hierarchical clustering and filtered out (as they had a lower number of counts compared to other samples) after WGCNA quality control, as suggested by the WGCNA authors. Accordingly, 194 animals and 11,996 genes were used to construct an undirected, signed network. Topological overlap measure (TOM) was computed from AM where TOM was converted to dissimilarity TOM. Based on TOM dissimilarity, we used the dynamic tree cut v.1.63.1 (Langfelder et al., 2008) to identify the modules as the branches of the resulting dendrogram. As parameters, the minimum size per module was set to 50 genes with a high sensitivity to split the clusters (deepSplit = 4). Genes with a similar expression pattern across samples were grouped into the same module and arbitrarily labeled by number.

Weighted Gene Co-expression Network Analysis was used for summarizing the obtained modules by a concept of eigengene. Eigengenes are the first principal component of the expression matrix for each module and represent the weighted average of expression profile for each module. Modules highly correlated were merged based on the ME dissimilarity threshold of 0.2 leading to the final set of modules for constructing the network.

### Trait Association Analysis and Module Selection

After the phenotypic data were mean-centered and scaled, a linear model was fitted to analyze the association between the expression profiles of the MEs and the phenotypes (Li et al., 2018). The model included the place of birth, the season of production, and animal’s age, according to the equation:

$$y_{ijkl}=\mu +C_{i}+G_{j}+A_{k}+T_{l}+\varepsilon_{ijkl}$$

Where:

*y_ijkl_*: is the expression level of the eigengene in each module (*n* = 23);

_*μ*_ : is the intercept of ME;

*C_i_* : is the fixed effect for the place of birth (three levels = CPPSE, IMA, NOHO);

*G_j_*: is the fixed effect for the season of production (three levels = 2009, 2010, 2011);

*A_k_* : is the covariate for the animal’s age, in days;

*T_l_* : is the trait observation for each animal;

*ε_ijkl_*: is the random residual effect associated with each observation.

Modules associated with at least two beef quality or mineral traits (*p* ≤ 0.05) were selected for further analyses.

### Pathway Over-Representation Analysis

Pathway analysis was performed using ClueGO version 2.5.1 to identify gene KEGG pathways over-represented in the selected modules (Bindea et al., 2009). Redundant terms were grouped based on the kappa score = 0.4 (Bindea et al., 2009). The *p*-value was calculated and corrected with a Bonferroni step down. Only pathways with a *p*-value (pV) *p* ≤ 0.05 were selected. These analyses were carried out based on the *B. taurus* annotation, and the network visualization was performed on Cytoscape version 3.6.1 (Shannon et al., 2003).

### Hub Gene Selection

Highly connected genes (hub genes) are supposed to be the main regulators in the network and have a pivotal biological role concerning the associated trait (Langfelder and Horvath, 2007, 2008). Hub genes in the associated modules were selected based on the module membership ≥ 0.8 (Langfelder and Horvath, 2008). Among them, hub genes partaking in over-represented biological pathways previously identified were retained. Moreover, over-representation pathway analysis including all hub genes was applied following the approach previously described.

### Integration of eQTL and Co-expression Modules

A list of eQTLs from the same population and dataset (Cesar et al., 2018) evaluated in this work was provided. The dataset included 1,268 *cis-* and 10,334 *trans-*eQTLs based on the association between 461,466 SNPs and the expression level of 11,808 genes from 192 animals. Since the eQTLs have a known effect on gene expression, the eQTLs that target the hub genes (MM ≥ 0.8) in the selected modules were evaluated. A Fisher’s exact test was applied to assess the module under/over-representation (FDR = 0.05).

## Results

**FIGURE 1:**
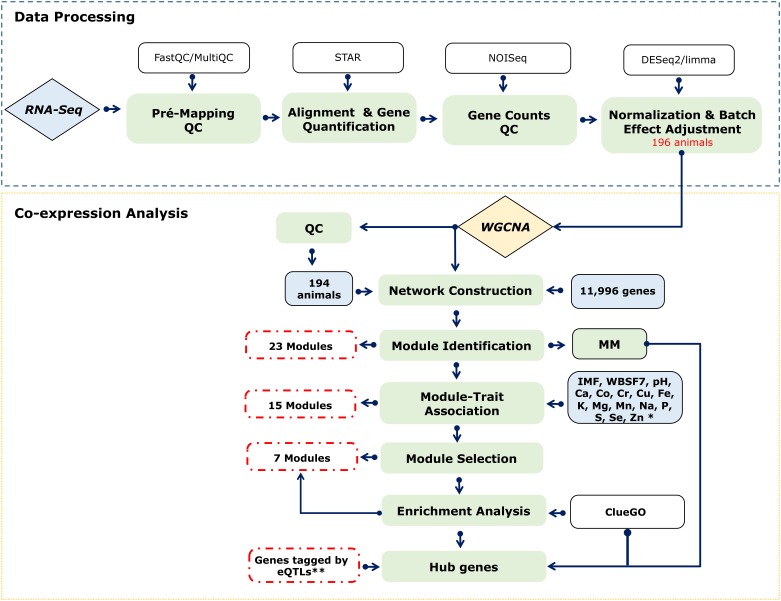
Workflow. Green boxes represent the main analysis steps that were involved in data processing and co-expression analysis. Tools applied in each step are shown in white boxes. Inputs and outputs are shown in blue and dashed red boxes, respectively. ^∗^A varying number of samples was analyzed for each trait (Supplementary Table S1). ^∗∗^Data from an eQTL analysis carried out for the same population used in this study (Cesar et al., 2018).

We applied a network-based approach to identify relevant genes and pathways associated with meat quality and mineral concentration in Nelore cattle (Figure 1). Based on the transcriptomic profiles of skeletal muscle samples of 194 steers, we constructed a signed weighted gene co-expression network with WGCNA (Langfelder and Horvath, 2008). From co-expressed modules and pathway analysis, we thereby identified several hub genes significantly associated with meat quality traits and mineral concentration.

### Descriptive Statistics and Correlation Estimates

**FIGURE 2:**
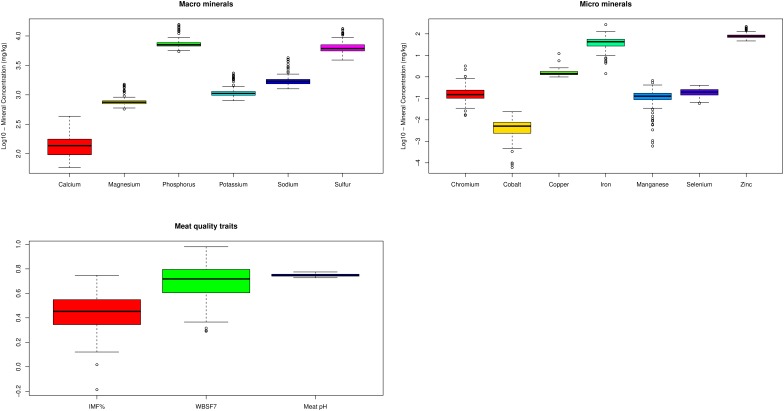
Box plot of mineral concentration (macro and micro) and meat quality traits. IMF% – Percentage of intramuscular fat content; WBSF7 – Warner-Bratzler Shear Force after 7 days of meat aging; The data are in *log10* scale.

We analyzed gene expression levels as measured with RNA-seq for association with three meat quality traits (intramuscular fat, meat pH, and tenderness) and the concentration of 13 minerals (Ca, Cr, Co, Cu, Fe, K, Mg, Mn, Na, P, S, Se, and Zn) available for a varying number of samples (ranging from 57 to 194, Supplementary Table S1). The genetic variance and heritability for the traits evaluated here, obtained from this population, ranged from low to moderate as previously published (Tizioto et al., 2013, 2015). A summary of descriptive statistics for each trait is in Supplementary Table S1 and Figure 2.

**FIGURE 3:**
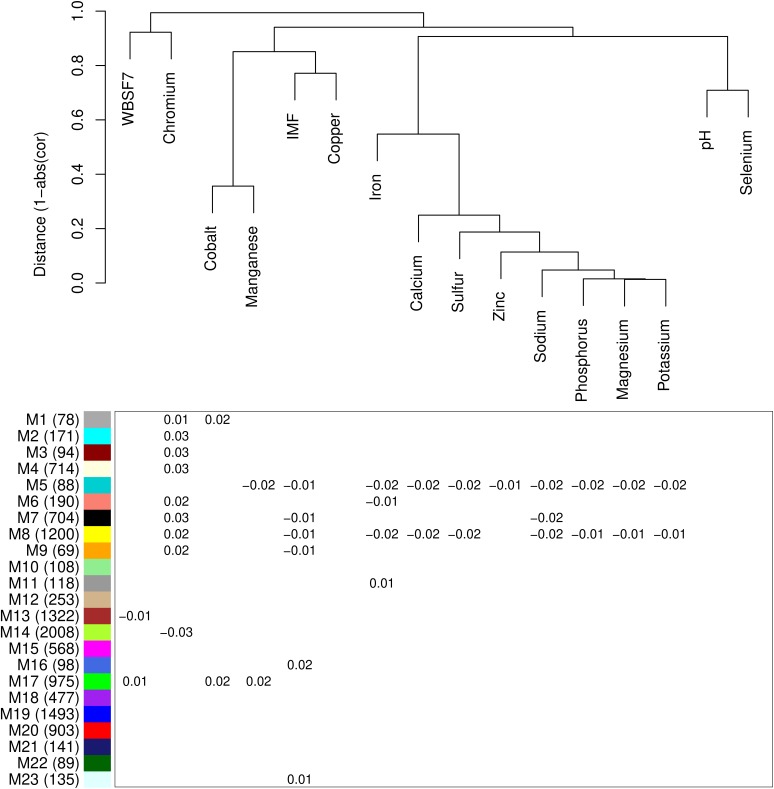
Hierarchical clustering of phenotypic correlation between traits (top) and module-trait association analysis (bottom). Modules are labeled by number on the *y*-axis with the number of contained genes in parenthesis. Each column represents a trait as indicated on the corresponding dendrogram branch. For significantly associated modules (*p* ≤ 0.05), the coefficient from the linear model is given within the cell.

We performed clustering analysis to identify similarities between traits (Figure 3 – top). We identified four clusters as follows: cluster 1 (WBSF7 and Cr), cluster 2 (Co, Cu, Mn, and IMF), cluster 3 (Fe, Ca, S, Zn, Na, P, Mg, and K), and cluster 4 (pH and Se). The pair-wise correlation within all traits is provided in Supplementary Figure S1. Significant and strong correlation ranged from 0.45 to 0.99 among minerals in the cluster 3 (*p* ≤ 0.05). We identified positive correlation among IMF with some minerals (Ca = 0.25, Cu = 0.23, Mn = 0.24, K = 0.17, Na = 0.3, S = 0.18, and Zn = 0.23) (*p* ≤ 0.05). Meat pH was positively correlated with Se (*r* = 0.29), whereas negatively associated with Fe (-0.17), Mg (-0.22), P (-0.25), K (-0.21), Na (-0.26), S (-0.17), and Zn (-0.22) (Supplementary Figure S1). No significant correlation was observed between tenderness (WBSF7), IMF, and meat pH.

### Data Processing and Co-expression Network Construction

On average, a total of 13 million of 100 bp paired-end reads per sample were generated. Around 96.71% of unique reads were mapped to the reference *B. taurus* genome (ARS-UCD1.2). Taking into account that low expressed genes are less reliable and indistinguishable from sampling noise (Tarazona et al., 2015), we filtered out the genes with less than one *cpm* in more than 90% of the samples. In addition, four samples were removed because they had a mapping rate lower than 70% or showed high variability on the housekeeping genes expression (see methods). Thus, we used 11,996 genes and 194 samples for the co-expression analysis.

Considering the WGCNA assumptions, the weighted network starts from the level of thousands of genes, identifies modules of co-expressed genes, summarizes the module expression profile as the first principal component (ME), and relates the MEs with the trait of interest (Langfelder and Horvath, 2008). The MM value quantifies the degree of co-expression of a gene with other genes within a module, thereby enabling the identification of intramodular hub genes.

From clustering 11,996 genes with WGCNA, we obtained 23 modules labeled by number (Figure 3). The module size ranged from 69 genes (M9) to 2,008 genes (M14) (Figure 3 – bottom). The proportion of variance explained by the eigengenes ranged from 0.18 (M20) to 0.53 (M5) (Supplementary Table S2).

### Trait Association and Pathway Enrichment Analysis

We performed an association analysis to identify the relationship between network and traits. This analysis measures the strength of the effect and the direction of the association between the module (eigengenes) and the trait. Thus, if the association is positive, it means the trait increases with increasing “eigengene expression” or vice-versa. We selected seven modules (M1, M5, M6, M7, M8, M9, and M17), associated with at least two traits (*p* ≤ 0.05) (Figure 3 – bottom) once we also want to point out shared pathways among traits. We found the highest number of significant associated modules between M5 (ten associations; negative with IMF, and the concentration of Mn, Fe, Ca, S, Zn, Na, P, Mg, and K), followed by M8 (nine associations; positive with Cr, negative with IMF, and the minerals of cluster 3, except Zn). The average expression profile of M17 module showed association with three traits (positive with WBSF7, Co, and Mn) along with M7 (negative with Na and IMF, and positive with Cr concentration). For the modules M1, M6, and M9, we found an association with two traits. We identified a positive association among M6 and M9 with Cr concentration while a negative association was observed between M9 with IMF, and M6 with Fe concentration. M1 was positively associated with the concentration of Cr and Co. The modules with none or only one trait association were not included for further analysis.

The module membership values for all the genes for selected modules are given in Supplementary Table S3. We carried out a pathway over-representation analysis on ClueGo version 2.5.1 for the seven selected modules (Table 1 and Supplementary Table S4) to identify meaningful metabolic pathways involved with meat quality traits and mineral concentration. We detected several pathways (*p-*Value ≤ 0.05, group *p*-value corrected with Bonferroni step down) mainly related to energy and protein metabolism, such as AMPK and mTOR signaling pathways.

**Table 1:** Module characterization.

Module^a^	eQTLs^b^	TGE^c^	Hub genes^d^	Enriched pathways^e^
M1 (78)	13	8 (4)	(10) *RSAD2, LOC100139670, MX1, IFIH1, OAS1Y, DDX58, EIF2AK2, IRF7, LOC512486, IFI16*	• NOD-like receptor signaling pathway
M5 (88)	45	15 (3)	(9) *PTPRC, CTSS*, ***DOCK2***, ***IL10RA****, FERMT3, ITGB2*, ***LCP2****, CORO1A, PTPRJ*	• Phagosome • Cell adhesion molecules (CAMs)
M6 (190)	89	20 (6)	(20) *GNAI1, PLIN1, FABP4, PPP1R1B, PCK2, ADIPOQ*, ***PDE3B****, TKT, MGST1, LIPE, ELOVL6, MRAS, ACACA, GNG2, HACD2*, ***FASN****, FBP1*, ***ELOVL5****, PCK1, G6PD*	• Adipocytokine signaling pathway • AMPK signaling pathway
M7 (704)	276	76 (2)	(11) *CAV1, COL4A2, GNAI2, SEPT9, TNFRSF1A, YWHAB, COL4A1, TMSB4X, SPTAN1, MSN, PARVA*	• Ras signaling pathway • Focal adhesion
M8 (1,200)	414	129 (5)	(10) *COL1A2, DCN, FN1, COL1A1, FGFR1, COL3A1, CD44, ITGB5, DSE, CTSK*	• Glycosaminoglycan degradation • Steroid biosynthesis
M9 (69)	16	9 (2)	(1) *JUND*	• TGF-beta signaling pathway • ∙ Osteoclast differentiation
M17 (975)	126	66 (2)	(21) *ASH1L, BIRC6, MED13, HERC1, MED13L, KMT2A, HUWE1, KMT2C, ATM, HERC2, KMT2D, MED1, GNAQ, SMC1A, TPR, NSD1, UBR5, ARHGAP5, KAT6A, PRDM2, ITCH*	• Apoptosis • mTOR signaling pathway
**Total – 3304**	**979^f^**	**323 (24)**	**82**	


*The table shows hub genes and eQTL information for each module found to be significantly associated with two or more traits in Figure 3. ^*a*^Selected modules with the number of contained genes in parenthesis. ^*b*^eQTLs – Number of eQTLs associated with genes in a module (Based on Cesar et al., 2018); ^*c*^TGE – Number of module genes associated with eQTLs. In the parenthesis are the number of genes with a MM ≥ 0.8; ^*d*^Selected hub genes based on pathway analysis and MM; Hub genes associated with eQTLs are in bold; ^*e*^Pathways from module over-representation analysis taking all genes into the module (Supplementary Table S4) Group *p*-Value ≤ 0.05; ^*f*^760 unique eQTLs identified.*

### Hub Gene Selection, Pathway Analysis, and Integration With eQTLs

Highly connected genes are likely to play an important role both in the network’s topology and biological pathways. In this way, we combined a pathway-based gene analysis for each selected module (Supplementary Table S4) and gene connectivity measure (MM ≥ 0.8) (Supplementary Table S3) selecting 82 hub genes (Table 1, see methods). Further, taking advantage of an eQTL study carried out in the same population (Cesar et al., 2018), we screened whether the genes in the modules were underlying eQTLs, and applied a Fisher’s exact test to assess the module over-representation. We identified 323 genes targeted by 760 unique eQTLs (Table 1 and Supplementary Table S5) into the seven modules. In addition, we identified 24 out of 323 genes with a MM ≥ 0.8, and six of them are part of the hub gene list (Table 1). However, based on the Fisher’s exact test (FDR = 0.05) no significant over-/under-representation was detectable in these modules.

**FIGURE 4:**
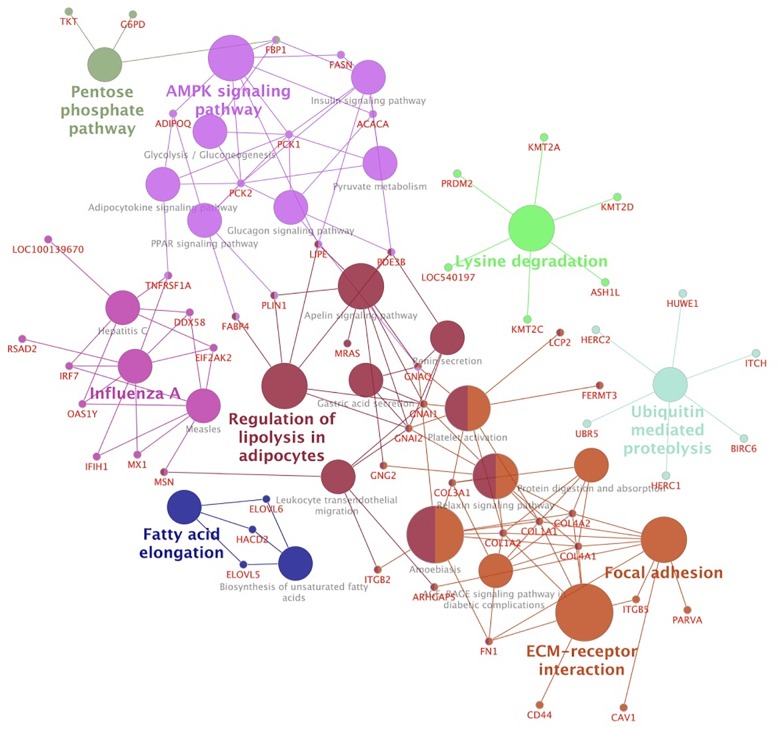
Network clusters based on over-represented KEGG pathways of hub genes associated with mineral concentration and meat quality traits. Functionally related groups partially overlap and are arbitrarily colored. The node size represents the pathway enrichment significance.

To gain further insights into their functions as well as to integrate the pathways among the modules, we carried out a KEGG pathway analysis. Considering a kappa score = 0.4 and *p-*Value ≤0.05 (Figure 4 and Supplementary Table S6), we clustered the identified pathways into eight groups. The pathways related to energy metabolism were clustered together and included AMPK, peroxisome proliferator-activated receptors (PPAR), insulin, glucagon, and adipocytokine signaling pathways. We also identified ubiquitin-mediated proteolysis and biosynthesis of fatty acids pathways over-represented in this network.

## Discussion

In this study, we analyzed genome-wide co-expression in skeletal muscle for association with mineral concentration and meat quality traits. Skeletal muscle metabolism is an integrated system dependent on the efficient coordination of gene expression, which are tightly regulated (Smith et al., 2013). We found several co-expression modules associated with two or more minerals, meat tenderness, and IMF, which indicates that common pathways influence these traits. From pathway analysis of module hub genes, we further found an over-representation for energy and protein metabolism (AMPK and mTOR). These pathways have been reported as the main drivers regulating energy balance in muscle (Smith et al., 2013). AMPK and mTOR are metabolically linked, nutritional and hormonal responsive, with an intricated relationship with insulin, thyroid hormone (TH), and TGF-beta signaling pathways (Xu et al., 2012), which were reported here as well. In addition, these pathways have been associated with muscle development, fat deposition, and beef quality traits (Du et al., 2009). Pathways related with muscle structure such as extracellular matrix, and focal adhesion, identified here, have also been identified in cattle co-expression networks (Reverter et al., 2006). The above-mentioned pathways are not the only ones acting on muscle metabolism. However, they showed an interaction with mineral concentration and meat quality in our study.

### Phenotype Correlation and Co-expression Network Analysis

In agreement with previous reports, we found several minerals positively correlated with IMF, but negatively correlated with meat pH. For instance, Cu-supplemented Angus were found with reduced back fat and reduced serum cholesterol level (Engle et al., 2000). Pigs supplemented with Mn showed an increased marbling and decreased pH consistent with the correlation identified here (Constantino et al., 2014). Furthermore, Se supplementation improved pork meat quality traits by increasing muscle pH (Calvo et al., 2017). In addition, these studies reported a positive effect against lipid oxidation. On the other hand, reduced levels of IMF were associated with low Zn concentration in lambs (Pannier et al., 2014).

Co-expression analysis resulted in 23 modules from which we considered seven modules for further analysis based on their association with at least two traits. The genes in modules like M5, M7, M8, and M9 were associated with IMF and several minerals suggesting a certain extent of co-regulation. It’s well known that minerals are essential in a wide range of biological processes. Here, we provide evidences that mineral content and meat quality traits are interrelated, as well as interplay with specific genes and pathways (as discussed below).

Variation in eQTL loci can explain a substantial fraction of variation observed on the gene expression level (Wang and Michoel, 2016). It has been observed that variation in eQTL loci is associated with concerted expression changes of many genes in co-expression clusters, thereby also impact the phenotype. Screening the detected co-expression modules, we found 323 genes affected by at least one eQTL. Despite 132 eQTLs targeting more than one module, most of the eQTLs were module-specific. However, no significant over-/under-representation (Fisher’s exact test) was detectable in these modules, suggesting that other regulatory mechanisms are involved. Despite that, the expression level of six hub genes was found affected by *trans-*eQTLs. These genes are involved with lipid metabolism [fatty acid synthase (*FASN*), and ELOVL fatty acid elongase 5 (*ELOVL5*), phosphodiesterase 3B (*PDE3B*)], immune system [lymphocyte cytosolic protein 2 (*LCP2*), and interleukin 10 receptor subunit alpha (*IL10RA*)], and actin remodeling (dedicator of cytokinesis 2 – *DOCK2*).

### Pathway Analysis

Over-representation pathway analysis in the selected modules (Table 1 and Supplementary Table S4) yielded glycosaminoglycan biosynthesis and degradation, lysosome, and steroid biosynthesis in the M8 module. Phagosome, cell adhesion molecular pathways, and NOD-like receptor signaling pathway were found enriched in M1 and M5. For the M17, enriched pathways included protein synthesis pathways such as mTOR, PI3K-Akt, TH, and AMPK signaling. We also found protein degradation pathways enriched in the M17 module such as ubiquitin-mediated proteolysis. TGF-beta signaling and osteoclast differentiation were enriched in the M9 module. Energy metabolism pathways were found enriched in M6, including glycolysis, fatty acid biosynthesis, AMPK, and insulin signaling. Ras, PI3K-Akt signaling pathways, and protein processing were found enriched in M7.

We also carried out cross-module enrichment analysis considering all hub genes, which indicated that the AMPK signaling pathway plays an important role for muscle mineral metabolism and meat quality traits. The genes of the AMPK pathway were also associated with IMF, Cr, and Fe. Furthermore, the AMPK pathway was also found enriched in genes of M17 (associated with WBSF7, Co, and Mg) and M6 (associated with Cr and Fe concentration).

#### Energy and Lipid Metabolism

AMP-activated protein kinase signaling is a major regulator of the cellular energy status, protein metabolism, and muscle metabolism (Je et al., 2006; Du et al., 2009; Mihaylova and Shaw, 2011). We found carbohydrate and fatty acid metabolism connected by the AMPK pathway (Figure 4). Hub gene *ACACA* was thereby involved in pyruvate metabolism, glucagon and insulin signaling pathways. Co-expressed in the M6 module, *ACACA* and *FASN* encode rate-limiting enzymes for long-chain fatty acid synthesis (Mihaylova and Shaw, 2011; Ropka-molik et al., 2017). *ACACA* catalyzes malonyl-CoA from acetyl-CoA, which is a substrate for the *FASN* enzyme in *de novo* fatty acid synthesis (Menendez and Lupu, 2007; Du et al., 2009). These genes, as well as fatty acid binding protein 4, adipocyte (*FABP4*), are regulated by the thyroid hormone responsive gene (*THRSP*) (Graugnard et al., 2009; Loor, 2010; Oh et al., 2014).

The co-expression of these genes, as well as the negative association between Fe concentration and lipid metabolism, were reported in our previous RNA-Seq work where *FASN, THRSP*, and *FABP4* were shown to be downregulated in animals with low Fe concentration in muscle (Diniz et al., 2016). Hay et al. (2016) reported a major role of Fe for lipid oxidative metabolism based on the downregulation of peroxisome proliferator-activating receptor gamma coactivator 1*α (PPARG1A)* measured by qRT-PCR. TH is also essential for energy metabolism regulation, and Fe deficiency was found to impair TH synthesis and its regulatory function (Cunningham et al., 1998). Adipogenic genes are responsive to *PPARG* and *TH* (Graugnard et al., 2009). Thus, reduced adipogenesis has been associated with Fe deficiency (Cunningham et al., 1998; Diniz et al., 2016; Hay et al., 2016).

In addition to factors that increase the intracellular cyclic AMP level (Omar et al., 2009), Cr increases AMPK activity and positively affects the insulin sensitivity in skeletal muscle cells (Hoffman et al., 2015). As part of the insulin pathway, we found phosphoenolpyruvate carboxykinase 1 and 2 (*PCK1, PCK2*), fructose-bisphosphatase 1 (*FBP1*), and phosphodiesterase 3B (*PDE3B*), major regulators of glycolysis and gluconeogenesis (Pilkis and Granner, 1992). The *PDE3B* enzyme is stimulated by insulin and cAMP (Degerman et al., 2011) and affects the activation of AMPK (Omar et al., 2009). AMPK activation inhibits fatty acid synthesis and gluconeogenesis via repression of *ACACA* and *PCK*, respectively (Hardie, 2011). Unlike Fe, the concentration of Cr showed a positive correlation with M6. These minerals may have an antagonistic relationship (Staniek and Wójciak, 2018). However, the correlation between Fe and Cr concentration was not significant in this study most likely due to the limited sample size for Cr concentration.

Supplementing goats with Cr decreased the expression level of *ACACA, FASN*, and *FABP4* (Sadeghi et al., 2015) as measured by RT-PCR. Furthermore, increased *Longissimus* muscle area and reduced fat thickness was associated with a downregulation of *ACACA* expression in Cr-supplemented goats (Najafpanah et al., 2014). It seems to follow that Cr supplementation can improve meat quality by altering the direction of energy accumulation from fat deposition toward muscle growth in goats (Najafpanah et al., 2014; Sadeghi et al., 2015). Cr-supplemented Angus-cross steers were also found with increased *Longissimus* muscle area and decreased IMF without affecting growth performance (Kneeskern et al., 2016). Similar results were reported for Cr-supplemented pigs which showed lower backfat thickness and fat percentage (Pamei et al., 2014).

#### Muscle Development, Structure, and Proteolysis

As part of the TGF-beta signaling pathway, we identified the transforming growth factor beta 3 (*TGFB3*), which is involved in muscle proliferation, differentiation, and growth (Nishimura, 2015). However, muscle hypertrophy results from a balance of protein turnover in which AMPK signaling negatively affects the protein synthesis (Du et al., 2009). AMPK signaling also acts on cytoskeletal dynamics (Mihaylova and Shaw, 2011). As pointed out in Figure 4, common genes act on focal adhesion and ECM-receptor interaction. For these pathways, we found members of the collagen gene family (*COL1A1, COL1A2, COL3A1, COL4A1*, and *COL4A2*), glycoproteins and proteoglycans such as fibronectin 1 (*FBN1*) and decorin (*DCN*), respectively. These molecules are structural components of the ECM and are thus critical for muscle development (McCormick, 2009; Nishimura, 2015). These genes were also found associated with meat quality traits such as tenderness and IMF (Ponsuksili et al., 2013; Cesar et al., 2015; Nishimura, 2015). Except for *COL4A1* and *COL4A2*, all collagen genes reported above and which we found co-expressed in M8 were associated with the concentration of Ca, Cr, Fe, K, Mg, Na, P, S, and IMF. Tajima et al. (1981) reported that hypocalcemic fibroblast cells showed an increased synthesis of collagen. Fe concentration has also been associated with collagen metabolism due to the iron-dependent enzymes involved in collagen synthesis (Cammack et al., 1990).

We found ubiquitin-mediated proteolysis enriched across modules as well as for genes in the M17 (Supplementary Table S3), which was associated with WBSF7, Co, and Mn. Proteolytic enzymes are important for protein turnover and postmortem meat aging (Koohmaraie et al., 2002; Gonçalves et al., 2018). Baculoviral IAP repeat containing 6 (*BIRC6*) is a caspase inhibitor and apoptotic suppressor protein (Verhagen et al., 2001). *BIRC6* is part of the ubiquitin-mediated proteolysis pathway and was positively associated with M17. By impairing proteolysis, the up-regulation of *BIRC6* likely increases shear force (Liu et al., 2016). Genes from the E3 ubiquitin-protein ligase family (*HERC1, HERC2, HUWE1, ITCH*, and *UBR5*) were also identified in agreement with a recent report that found ubiquitination and apoptosis to be potential regulators of meat tenderness in Nelore cattle (Gonçalves et al., 2018).

## Conclusion

We demonstrated transcriptional relationships among mineral concentration and meat quality traits in the skeletal muscle of Brazilian Nelore cattle. We identified 82 hub genes across seven co-expression modules which seem to be critical for this interplay. The AMPK and mTOR signaling pathways were hereby found to link mineral and muscle metabolism in Nelore cattle. Future studies investigating different levels of mineral supplementation, the mineral interaction, and their effect in the gene expression and meat quality traits could help us to elucidate the regulatory mechanism by which the genes/pathways are affected.

## Data Availability

All relevant data are within the paper and its Supporting Information files. All sequencing data is available in the European Nucleotide Archive (ENA) repository (EMBL-EBI), under accession PRJEB13188, PRJEB10898, and PRJEB19421 (https://www.ebi.ac.uk/ena/submit/sra/). All additional datasets generated and analyzed during this study may be available upon request from the corresponding author on reasonable request.

## Author Contributions

WD, PT, LR, LC, and HK conceived the idea of this research. WD, GM, LG, FB, and AC, JA, PdO, PT carried out the bioinformatics and data analysis. WD, GM, PB, FB, HK, JA, LC, LR collaborated with the interpretation of results, discussion and review the manuscript. WD, PB, and GM drafted the manuscript. All authors have read and approved the final manuscript and agreed to be accountable for the content of the work.

## Conflict of Interest Statement

The authors declare that the research was conducted in the absence of any commercial or financial relationships that could be construed as a potential conflict of interest.

## Footnotes

1 https://github.com/ibest/seqyclean

2 https://www.bioinformatics.babraham.ac.uk/projects/fastqc/

3 http://multiqc.info/

## Supplementary Material

FIGURE S1 Correlation matrix of mineral concentration and meat quality traits in Nelore cattle. Each cell displays the correlation value when significant (*p* ≤ 0.05). The matrix is color-coded by correlation according to the color legend.

TABLE S1 Summary statistics of meat quality traits and mineral concentration in Nelore cattle.

TABLE S2 The proportion of variance explained by the module eigengene (MEs).

TABLE S3 Gene list and module membership for each selected module. Spreadsheet tabs are divided by module.

TABLE S4 Summary of pathway analysis from ClueGo for genes clustered into the selected modules. Spreadsheet tabs are divided by module.

TABLE S5 Genes targeted by eQTLs for each selected module. Genes with MM ≥ 0.8 are highlighted in bold. Spreadsheet tabs are divided by module.

TABLE S6 Summary of pathway analysis from ClueGo for hub genes.

## Abbreviations

AMPK: AMP-activated protein kinase
CPM: Counts *per* million
ECM: Extra Cellular Matrix
IMF: Intramuscular Fat Content
ME: Module eigengene
MM: Module Membership
QC: Quality Control
WBSF7: Warner-Bratzler Shear Force after 7 days of meat aging
WGCNA: Weighted Gene Co-expression Network Analysis
