# Supplementary figures and images for: Detection of Co-expressed Pathway Modules Associated With Mineral Concentration and Meat Quality in Nelore Cattle
Source: Front Genet. 2019 Mar 13;10:210. doi: 10.3389/fgene.2019.00210 (PMC6424907; doi:10.3389/fgene.2019.00210)

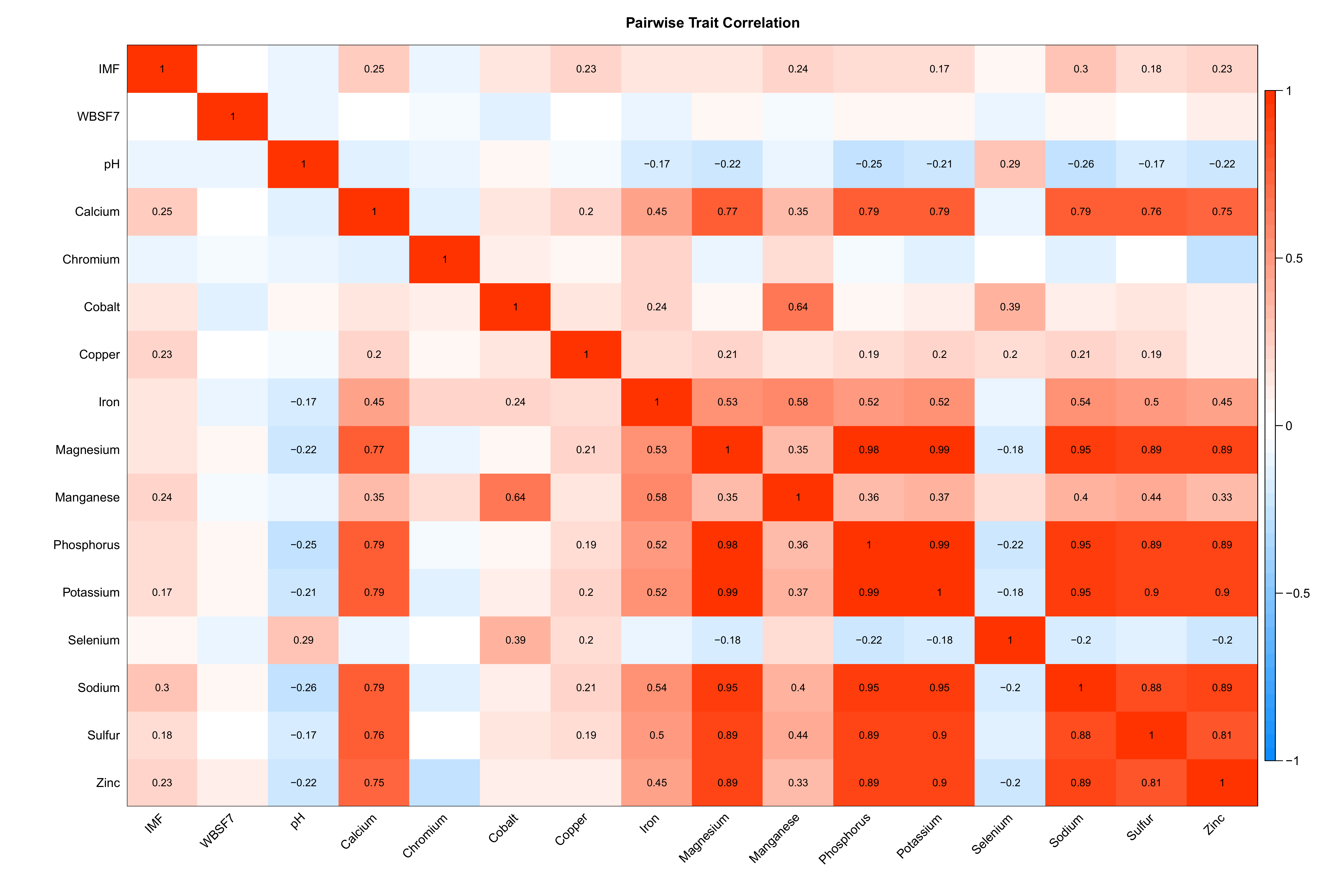

Supplement: FIGURE S1 — Correlation matrix of mineral concentration and meat quality traits in Nelore cattle. Each cell displays the correlation value when significant (p ≤ 0.05). The matrix is color-coded by correlation according to the color legend. [file Image_1.TIF]
